# IS*Ecp1*-associated dissemination of *bla*_CTX-M_/*bla*_CMY-2_ among cefotaxime-resistant non-typhoidal *Salmonella* from patients with diarrhea in Chongqing, China, 2022–2024

**DOI:** 10.3389/fcimb.2026.1891482

**Published:** 2026-09-28

**Authors:** Na Zhao, Gang Duan, Yu Liu, Chunyan Liao, Ling Wang, Yu Zhao, Yanbin Zhou, Fei Chen, Caiyun Li, Tingyan Sun, Yong Zhu, Mansha Yuan, Linghan Kong

**Affiliations:** 1 Chongqing Wanzhou Center for Disease Control and Prevention, Chongqing, China; 2 Chongqing Municipal Center for Disease Control and Prevention, Chongqing, China; 3 Yuzhong District Center for Disease Control and Prevention, Chongqing, China; 4 Chongqing Shapingba Center for Disease Control and Prevention, Chongqing, China; 5 Chongqing Nanan Center for Disease Control and Prevention, Chongqing, China; 6 Chongqing Yongchuan Center for Disease Control and Prevention, Chongqing, China; 7 Chongqing Rongchang Center for Disease Control and Prevention, Chongqing, China

**Keywords:** *bla_CTX-M_/bla_CMY-2_*, CTX-resistant, IS*Ecp1*, non-typhoidal *Salmonella*, plasmid

## Abstract

**Background:**

CTX-resistant Salmonella infections threaten public health. This study aimed to characterize the genetic environments of *bla*_CTX-M_/*bla*_CMY-2_ in CTX-resistant non-typhoidal *Salmonella* from patients with diarrhea in Chongqing, China (2022–2024).

**Methods:**

Among 306 *Salmonella* isolates recovered from seven hospitals, 36 (11.8%) were confirmed as CTX-resistant. Antimicrobial susceptibility testing, whole-genome sequencing (WGS), genetic structure analysis, conjugation experiments, and core-genome single nucleotide polymorphism (cgSNP) analysis were performed.

**Results:**

All 36 CTX-resistant isolates were resistant to CTX and ampicillin; resistance rates to chloramphenicol (88.9%), tetracycline (86.1%), and ceftazidime (63.9%) were high. Thirteen serotypes were identified, with *S*. I 4,[5],12:i:- (30.6%), *S.* Goldcoast (16.7%), and *S.* Typhimurium (11.1%) being the most frequently identified. The most frequently detected ESBL genes were *bla*_CTX-M-65_ (17/36) and *bla*_CTX-M-55_ (11/36). *bla*_CMY-2_ occurred in five isolates. *QnrS* co-carriage was 72.2% (26/36). IS*Ecp1* flanked *bla*_CTX-M_/*bla*_CMY-2_ in 94.4% (34/36) of isolates, highlighting its major role in mobilizing these resistance genes. IncHI2A was the most frequently identified plasmid (50.0%). In an *S*. I 4,[5],12:i:- isolate, a 96,117-bp IncI1 plasmid carrying IS*Ecp1*-*bla*_CMY-2_ was 100% identical to a Botswana *E. coli* plasmid. In an *S*. Thompson isolate, a 152,834-bp IncC plasmid carrying the same transposition unit shared >99% identity with a plasmid from a Hefei clinical isolate. Notably, a novel 75,302-bp IS*Ecp1*-mediated transposition unit carrying *bla*_CTX-M-65_, together with the entire IncI plasmid backbone, was identified with 5-bp direct repeats. This unit is far larger than typical IS*Ecp1*-mediated insertions (2.9–3.0 kb).

**Conclusion:**

Our findings suggest that ISEcp1 is associated with the dissemination of *bla*_CTX-M_/*bla*_CMY-2_ among CTX-resistant isolates in this regional collection, and suggest the potential to mobilize large DNA segments. Enhanced genomic surveillance of such mobile elements is urgently needed in this region.

## 1 Introduction

Non-typhoidal *Salmonella* (NTS) is a major cause of bacterial gastroenteritis worldwide. Infections from *Salmonella* have occurred worldwide, Typhoidal and NTS affect more than 150 million people worldwide annually (Giorgio and Helaine, 2025). In the United States, antimicrobial-resistant (AMR) NTS alone accounts for approximately 212,500 infections and 90 deaths *Salmonella* is recognized as one of the leading causes of bacterial foodborne illness each year, according to the 2019 AR Threats Report (CDC, 2019). In China, illnesses are often caused by animal-origin foods serving as key transmission vectors (Li et al., 2025, 2020a).

Antimicrobial therapy is essential for treating invasive *Salmonella* infections, particularly in children, the elderly, and immunocompromised patients. Third-generation cephalosporins, such as ceftriaxone (CRO) and cefotaxime (CTX), have long served as first-line agents for empirical treatment of suspected *Salmonella* infections. In the past few decades, the acquired gene *bla*_CTX-M_, as the most common extended-spectrum β-lactamase (ESBL) gene, has been widespread globally and its prevalence continues to increase (Wang et al., 2025b). The World Health Organization (WHO) has designated fluoroquinolone-resistant and third-generation cephalosporin-resistant *Salmonella* as high-priority pathogens in its 2024 Bacterial Priority Pathogens List, particularly noting their high burden in low- and middle-income countries (WHO, 2024).

The dissemination of the cephalosporin resistance genes *bla*_CTX-M/_*bla*_CMY_ is primarily driven by mobile genetic elements, particularly insertion sequences and plasmids. The insertion sequence IS*Ecp1* plays a critical role in capturing, mobilizing, and enhancing the expression of *bla*_CTX-M_ genes (Wang et al., 2025a; Cantón et al., 2012). A large-scale analysis of 1,393 cephalosporin- resistant *Shigella* isolates collected in China (2004-2016) revealed that the carriage rate of IS*Ecp1* was 90.09%, and the IS*Ecp1*-*bla*_CTX-M_ element was detected in 67.19% of isolates (Zhang et al., 2025). Plasmids of different incompatibility groups serve as the primary vectors for the global dissemination of these resistance genes (Zamudio et al., 2024). IncI1 plasmids are common among *E. coli* and *Salmonella* from poultry and other avian sources, and have been increasingly recognized as important vectors in clinical isolates from human diarrheal infections (Foley et al., 2021). In Shenzhen, *bla*_CTX-M_/*bla*_CMY-2_-carrying contigs in clinical *Salmonella* isolates were located on IncI1, IncK1, or IncA/C plasmids, with IS*Ecp1* associated with these resistance genes (Wang et al., 2025a). Together, these findings suggests that IS*Ecp1* and diverse plasmid types act in concert to facilitate the capture, expression, and horizontal spread of *bla*_CTX-M_/*bla*_CMY-2_ among *Salmonella* and other Enterobacteriaceae.

Although the prevalence of ESBL and AmpC genes in *Salmonella* has been widely reported, comprehensive characterization of the genetic environments of *bla*_CTX-M_/*bla*_CMY-2_ in CTX-resistant *Salmonella* isolates from patients with diarrhea in Chongqing remains limited. Specifically, the distribution of IS*Ecp1* elements, the types of plasmids carrying these resistance genes, and the potential for large-scale mobile genetic elements (MGEs) mediated events have not been systematically investigated in this region. This study therefore characterized these genetic contexts in 36 CTX-resistant *Salmonella* isolates collected from seven hospitals in Chongqing (2022–2024). Using antimicrobial susceptibility testing, serotyping, multilocus sequence typing (MLST), and whole-genome sequencing (WGS), we elucidated the genetic contexts, mobile genetic elements, and plasmid types associated with these resistance genes. Our findings provide insights into the dissemination mechanisms of CTX-resistant *Salmonella* in Chongqing and highlight the need for enhanced surveillance and infection control in this region.

## 2 Materials and methods

### 2.1 Ethical statement

The present study was reviewed and approved by the Ethics Review Committee of Chongqing Wanzhou Center for Disease Control and Prevention (Project No: WZCDC-HEC-2026001). The right and the welfare of the subject are adequately protected; the potential risks are outweighed by potential benefits.

### 2.2 Bacterial isolates

From 2022 to 2024, 306 *Salmonella* strains were isolated from fecal samples of patients with diarrhea at seven hospitals in Chongqing. The collected samples (2–3 g for feces; 1–3 mL for other liquid samples) were inoculated onto *Salmonella-Shigella* (SS) agar plates using a standardized streaking technique. The plates were then incubated aerobically at 37°C for 24 hours. Following incubation, a single presumptive *Salmonella* colony was selected from each plate. The species of isolate was confirmed by Matrix-assisted Laser Desorption/Ionization Time-of-Flight Mass Spectrometry (MALDI-TOF MS). Colonies confirmed as *Salmonella* spp. were subsequently streaked onto xylose lysine deoxycholate (XLD) agar plates for purification.

### 2.3 Antimicrobial susceptibility testing

Antimicrobial susceptibility was determined by a Customized AST plate CHNENF (Thermo, West Sussex, UK) for the following antimicrobials: chloramphenicol (CHL, 4–32 μg/mL), trimethoprim-sulfamethoxazole (SXT, 0.5/9.5–8/152 μg/mL), colistin (CT, 0.25–8 μg/mL), ertapenem (ETP, 0.25–8 μg/mL), meropenem (MEM, 0.12–2 μg/mL), cefotaxime (CTX, 0.25–16 μg/mL), ceftazidime (CAZ, 0.25–16 μg/mL), ceftazidime/avibactam (CZA, 0.25/4–8/4 μg/mL), tetracycline (TET, 1–16 μg/mL), tigecycline (TGC, 0.25–8 μg/mL), ciprofloxacin (CIP, 0.015–2 μg/mL), nalidixic acid (NAL, 4–32 μg/mL), amikacin (AMI, 4–64 μg/mL), streptomycin (STR, 8–32 μg/mL), ampicillin (AMP, 2–32 μg/mL) and ampicillin/sulbactam (AMS, 2/1–32/16 μg/mL). The minimum inhibitory concentration (MIC) was interpreted using the Clinical and Laboratory Standards Institute (CLSI M100, 2022) guidelines. *E. coli* ATCC25922 was used for microbiological quality control.

### 2.4 Whole genome sequencing and analysis

Genomic DNA was extracted using the bacterial genomic DNA extraction kit (TianGen, Beijing, China) following the standard protocol. DNA libraries were constructed using a Rapid Plus DNA Lib Prep Kit (ABclonal, Woburn, United States) and sequenced on an Illumina PE150 (Illumina Inc., San Diego, CA, United States) to generate 2 × 150-bp paired-end reads. Raw reads were trimmed for primers, adaptors and unqualified reads using fastp v0.22.0 (Chen et al., 2018). The filtered reads were assembled by SPAdes v3.0 using default parameters with the “–careful” option (Bankevich et al., 2012). The *Salmonella* serotypes were predicted using the SeqSero2 (Li et al., 2020b). Multilocus sequence typing (MLST) with a cut-off of 90% identify and 80% coverage were analyzed using MLST 2.0. ARGs were identified using ResFinder 4.7.2 (https://cge.food.dtu.dk/services/ResFinder/, accessed in 2025). The following parameters were applied, for identity threshold ≥95.00%, minimum length coverage ≥90.00%. To validate the reliability of the annotations, key resistance genes (*bla*_CTX-M_/*bla*_CMY-2_) were cross-validated using the Basic Local Alignment Search Tool (BLAST) from the National Center for Biotechnology Information (NCBI). The phylogenetic tree was constructed using MStree2 and subsequently visualized with iTOL v6 (Letunic and Bork, 2024). Contigs carrying *bla*_CTX-M_/*bla*_CMY-2_ genes were retrieved from the draft genomes and analyzed using the ISfinder platform (https://tncentral.ncc.unesp.br/ISfinder/about.php) to identify insertion sequences, PlasmidFinder 2.1 (https://cge.food.dtu.dk/services/PlasmidFinder/) to detect plasmid replicons, and BLAST (https://blast.ncbi.nlm.nih.gov/Blast.cgi) for sequence homology and annotation. Easyfig (http://mjsull.github.io/Easyfig/) was used to compare the different MGEs.

### 2.5 Conjugation experiments

Conjugation experiments were performed using cefotaximeresistant *Salmonella* strains as donors and *E. coli* C600 (which exhibits high-level rifampin resistance) as the recipient. Donor and recipient strains were inoculated in 2 mL of LB broth and incubated at 37 °C, 180 rpm for 4 hours. The cultures were then mixed in a 1:4 ratio (v/v) and incubated at 37 °C for 12 h. Transconjugants were selected on SS agar plates containing cefotaxime (20 μg/mL) and rifampin (400 μg/mL) and confirmed by PCR detection of *bla*_CTX-M/_*bla*_CMY_ (Wang et al., 2025a). All experiments were conducted in triplicate.

### 2.6 Core-genome single nucleotide polymorphisms analysis

Phylogenetic analysis of the same MLST or serotype strains based on cgSNPs was performed using CSI Phylogeny 1.4 (Kaas et al., 2014), with the default parameters. As previously described (Kudirkiene et al., 2020), a cut-off value of <5 cgSNPs was applied to identify closely related strains, and visualized by Graphtree (Zhou et al., 2018).

### 2.7 Genomes accession numbers

The whole-genome sequences of the 36 *Salmonella* isolates analyzed in this study have been deposited in the NCBI database, and the BioSample accession numbers are listed in **Supplementary Data 1**.

## 3 Results

### 3.1 Antimicrobial susceptibility profile

A total of 36 (11.8%) CTX-resistant *Salmonella* isolates were obtained from seven hospitals in Chongqing, China, between 2022 and 2024. Antimicrobial resistance phenotypes of the strains are detailed in **Supplementary Data 1**. Among these isolates, all 36 strains exhibited resistance to CTX and AMP. Furthermore, the resistance rates of other antimicrobial agents were as follows: CHL 88.89% (32/36), TET 86.11% (31/36), CAZ 63.89% (23/36), AMS 61.11% (22/36), SXT 52.78% (19/36) and CIP 25% (9/36), **Table 1**. No strains were found to be resistant to CT, CZA, ETP, MEM, TGC or AMI. The MICs for the 36 strains were also determined (see **Supplementary Data 1**).

**Table 1:** Antimicrobial susceptibility profiles of *Salmonella* strains.

Antimicrobial agent	No. of resistant isolates (prevalence, %)
Phenicols	
CHL	32, 88.89%
Folate pathway inhibitors	
SXT	19, 52.78%
Lipopeptide	
CT	0
Penicillins	
AMP	36, 100.00%
β-lactam/β-lactamase inhibitors	
AMS	22, 61.11%
CZA	0
Cephems	
CTX	36, 100.00%
CAZ	23, 63.89%
Carbapenems	
ETP	0
MEM	0
Tetracyclines	
TET	31, 86.11%
TGC	0
Fluoroquinolones	
CIP	9, 25.00%
Aminoglycosides	
AMI	0

### 3.2 Molecular characteristics of CTX-resistant *Salmonella* isolates

A total of 13 serotypes were identified among the 36 CTX-resistant *Salmonella* isolates. Among these, *S.* I 4,[5],12:i:- (11/36, 30.6%) was the most frequently identified, followed by *S.* Goldcoast (6/36, 16.67%), *S.* Typhimurium (4/36, 11.11%), *S.* Thompson (3/36, 8.33%), *S.* Infantis (2/36, 5.56%), and *S.* Agona (2/36, 5.56%). The remaining serotypes (*S.* London, *S.* Bovismorbificans, *S.* Muenster, *S.* Rissen, *S.* Enteritidis, *S.* Kentucky and *S.* Montevideo) were each identified in one isolate (2.8%). MLST analysis identified all 36 CTX-RSE isolates as belonging to 13 STs (**Figure 1**).

**Figure 1:**
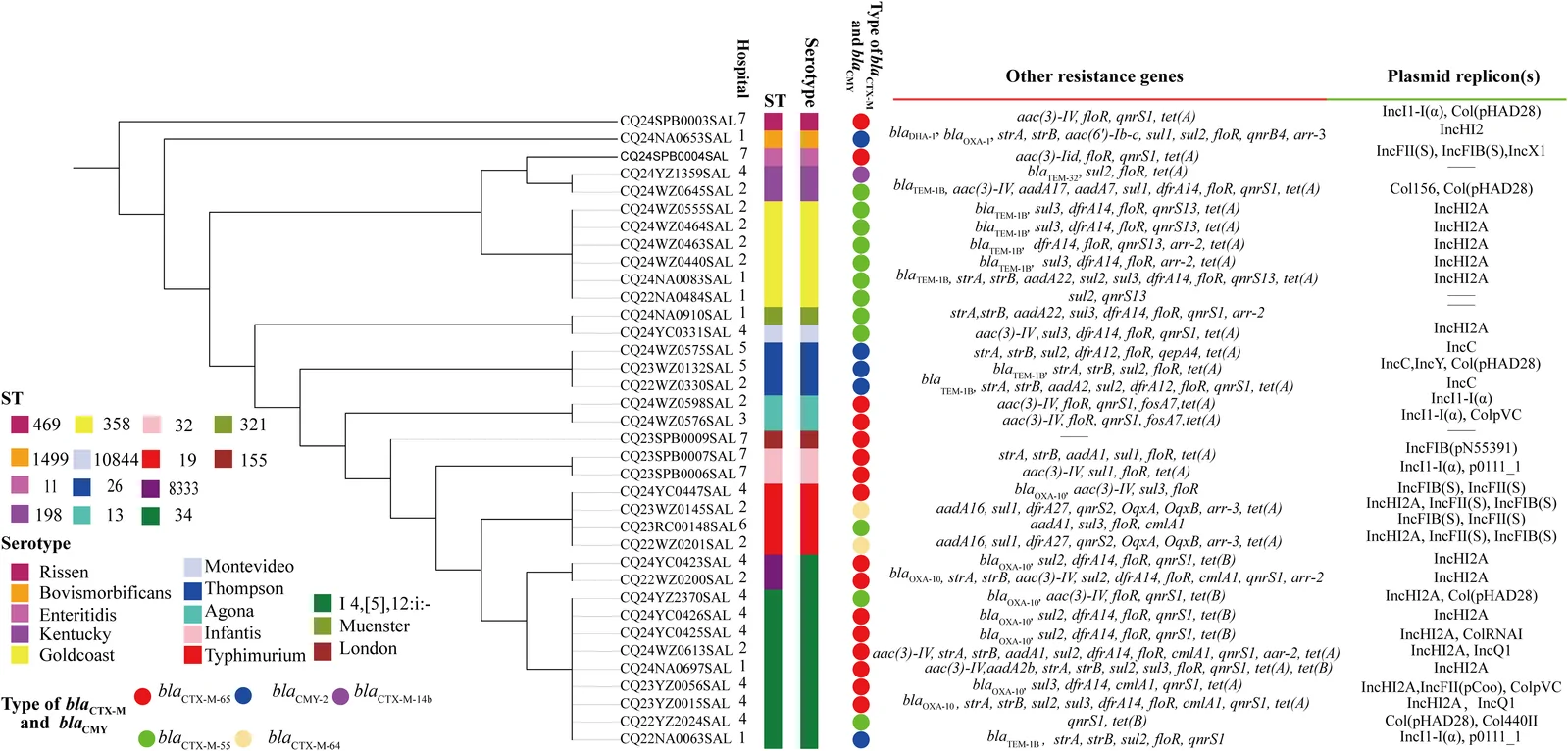
The MLST, serotypes, ARGs, and plasmids of 36 CTX-resistant *Salmonella* strains.

To investigate the genetic mechanisms underlying antimicrobial resistance, whole-genome sequences of all isolates were screened for resistance genes. A total of 37 different resistance genes were identified across the 36 *Salmonella* isolates, conferring resistance or reduced susceptibility to β-lactams, aminoglycosides, quinolones, tetracyclines, chloramphenicols, folate pathway inhibitors, fosfomycin, and rifampicin. ESBL genes *bla*_CTX-M_ (n=31) and AmpC β-lactamase genes *bla*_CMY-2_ (n=5) were detected. These genes were found to be distributed across 36 isolates. Four *bla*_CTX-M_ variants were detected: *bla*_CTX-M-65_ (n=17), *bla*_CTX-M-55_ (n=11), *bla*_CTX-M-64_ (n=2), and *bla*_CTX-M-14b_ (n=1) (**Figure 1**). AmpC β-lactamase genes *bla*_CMY-2_ were identified in three *S.* Thompson, one *S.* I 4,[5],12:i:-, and one *S.* Bovismorbificans. Other β-lactamase genes were also detected, including *bla*_TEM-1B_ (n=10), *bla*_OXA-10_ (n=8), *bla*_DHA-1_ (n=1), *bla*_TEM-32_ (n=1). Furthermore, the majority of CTX-resistant *Salmonella* isolates co-harbored other resistance genes, including sulfonamide resistance gene *sul* (n=32/36), phenicol resistance gene *floR* (n=30/36), quinolone resistance gene *qnrS/qnrB* (n=27/36), and tetracycline resistance gene *tet(A)* (n=23/36) (**Supplementary Data 2**; **Supplementary Table S1**).

Among the 36 -resistant *Salmonella* isolates, co-carriage of *bla*_CTX-M_ and *qnrS/qnrB* was detected in 27 isolates (75.0%). These isolates spanned 10 distinct serotypes, including *S.* I 4,[5],12:i:- (n=11), *S.* Goldcoast (n=5), *S.* Typhimurium (n=2), *S.* Agona (n=2), and one isolate each of *S.* Thompson, *S.* Bovismorbificans, *S.* Rissen, *S.* Enteritidis, *S.* Montevideo, and *S.* Kentucky (**Figure 1**).

A total of 16 incompatibility (Inc) groups were detected, with the IncHI2A (18/36, 50.0%), IncFII(S) (5/36, 13.9%), IncFIB(S) (5/36, 13.9%) and Col(pHAD28) (5/36, 13.9%) plasmids being the most frequently identified plasmid replicon types (**Figure 1**). A subset of isolates (4/36, 11.1%) contained no plasmid replicons.

### 3.3 Genetic structures of *bla*_CTX-M_/*bla*_CMY-2_ in CTX-resistant *Salmonella* isolates

The lengths of *bla*_CTX-M_/*bla*_CMY-2_-carrying contigs ranged from 2,375 to 76,767-bp (**Supplementary Data 2**; **Supplementary Table S2**). IS*Ecp1* was detected flanking *bla*_CTX-M_/*bla*_CMY-2_ in 94.4% (34/36) of the isolates. Two of these contigs were located on plasmids. However, for most contigs, replicon genes (4/36, 11.1%) or plasmid backbone (30, 83.3%) were absent. This was due to incomplete assembly and the presence of multiple insertion elements, making it difficult to determine their exact location.

Six different genetic environments of *bla*_CTX-M-55_ were identified in 12 *Salmonella* isolates (**Figure 2A**). In *S.* Muenster isolate CQ24NA0910SAL, a 2,981-bp transposition unit (IS*Ecp1*-*bla*_CTX-M-55_-Δ*orf477*) was inserted into the incomplete mobile element ΔTn2 (4117-bp), generating 5-bp direct repeat sequences (DRs, 5’-TCATA-3’). A 5,667-bp segment (ΔIS*Lbr1*-*qnrS1*-IS*Kpn19*) was observed downstream of the transposition unit. This *bla*_CTX-M-55_-bearing contig (12,838-bp) showed similarity (>99.99%) to the corresponding chromosomal region of the *S.* Muenster isolate PJM1 (CP045038.1), which was obtained from an animal in Guangzhou, China.

**Figure 2:**
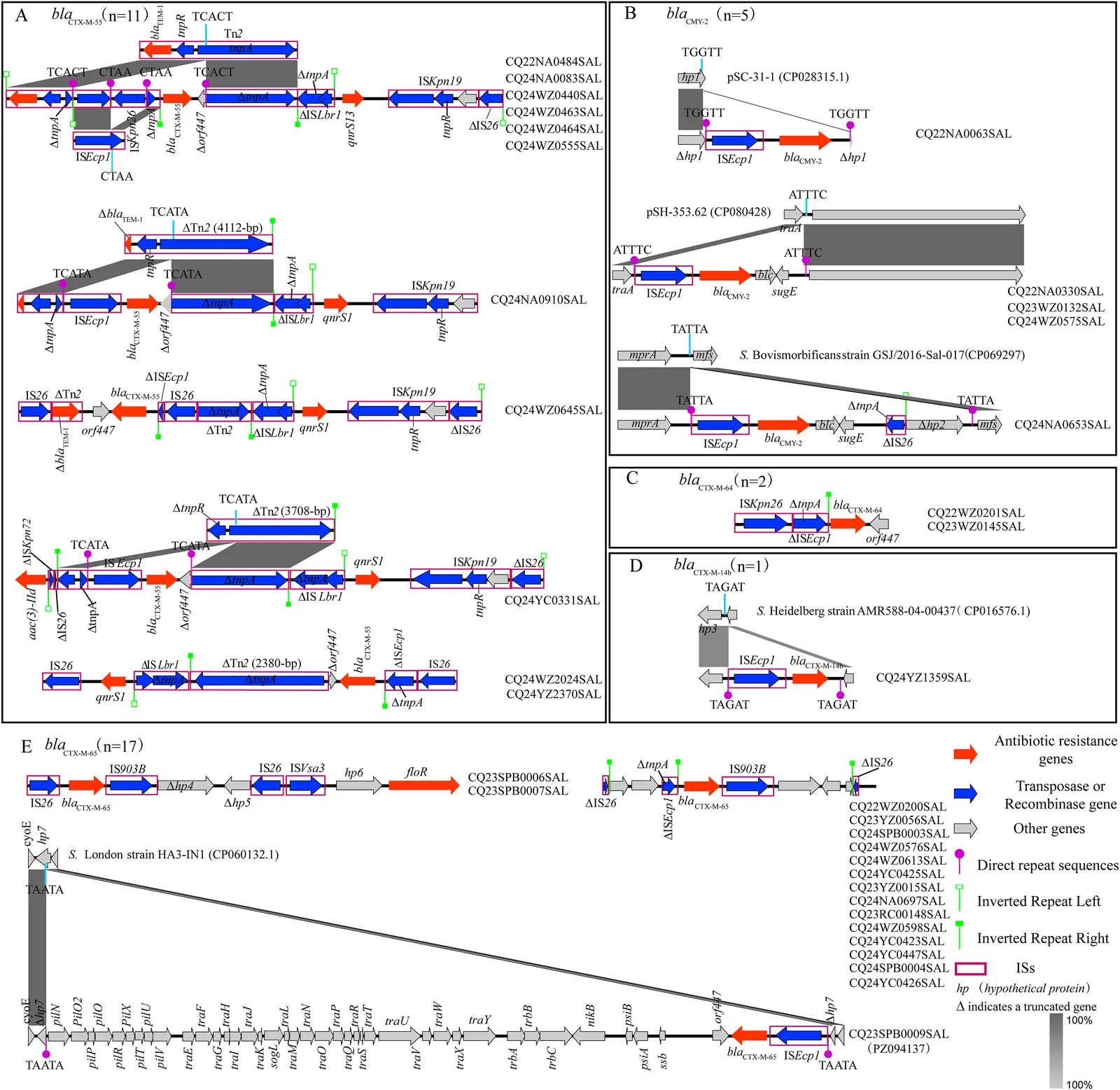
The genetic environments of *bla*_CTX-M_/*bla*_CMY-2_ in 36 *Salmonella* isolates in this study. **(A)** *bla*_CTX-55_; **(B)** *bla*_CMY-2_; **(C)** *bla*_CTX-M-64_; **(D)** *bla*_CTX-M-14b_; **(E)** *bla*_CTX-65_.

An identical transposition unit (IS*Ecp1*-*bla*_CTX-M-55_-Δ*orf477*) inserted into an incomplete mobile element ΔTn*2* (3708-bp) with matching 5-bp direct repeats (DRs, 5’-TCATA-3’) was detected in the *S.* Montevideo isolate CQ24YC0331SAL. A 5,979-bp segment (ΔIS*Lbr1*-*qnrS1*-IS*Kpn19*-ΔIS*26*) was observed downstream of this transposition unit and a 2,057-bp (*aac(3)-IId*-ΔIS*Kpn72*-ΔIS*26*) segment was observed upstream of this transposition unit. This *bla*_CTX-M-55_-bearing contig (13,864-bp) showed 78% coverage and 100% identity to the corresponding region of an IncHI2 plasmid, pAA738 (CP140756.1), from a *S.* 1,4,[5],12:i:- isolate obtained from a patient in Winnipeg, MB, Canada (**Supplementary Figure 1**).

In six *S.* Goldcoast isolates, the 2,981-bp transposition unit was inserted into a mobile element Tn*2*, generating 5-bp DRs (5’-TCACA-3’). Mobile element IS*Kpn26* was inserted into the IS*Ecp1* gene of this transposition unit with 4-bp DRs (5’-CTAA-3’). A 6,449-bp segment (ΔIS*Lbr1*-*qnrS13*-IS*Kpn19*-ΔIS*26*) was observed downstream of the transposition unit. This *bla*_CTX-M-55_-bearing contig (15,583 -bp) shared 100% identity with the corresponding region of the IncHI2 plasmid pSal-5364, which was identified in a *S.* Goldcoast isolate from a patient in Hangzhou, China.

A 6,718-bp segment (ΔIS*Ecp1*-*bla*_CTX-M-55_-Δ*orf477-*ΔTn*2*-ΔIS*Lbr1*-*qnrS1*) flanked by IS*26* was found in two *S.* I 4,[5],12:i:- isolates CQ24YZ2370SAL and CQ22YZ2024SAL. This *bla*_CTX-M-55_-bearing contig (8,993-bp) shared 100% identity with the corresponding chromosomal region of *S.* I 4,[5],12:i:- isolate L-4526 (AP023303.1), which was obtained from an animal in Japan.

In the *S.* Kentucky isolate CQ24WZ0645SAL, a 2767-bp segment (ΔIS*Ecp1*-*bla*_CTX-M-55_-Δ*orf477*-ΔTn*2*) was flanked by inverse-oriented IS*26* elements, and the commonly observed structure (ΔTn*2*-ΔIS*Lbr1*-*qnrS1*-IS*Kpn19*-ΔIS*26*) was found immediately upstream of this segment. This *bla*_CTX-M-55_-bearing contig (12,200 -bp) exhibited 100% similarity to the corresponding chromosomal region of two *S.* Kentucky isolates, Sal-FJ2064 (CP104049) and AH19MCS1 (CP102719.1), which were obtained from a clinical patient and from chicken meat products, respectively, in China.

Three different genetic environments of *bla*_CMY-2_ were identified in 5 *Salmonella* isolates (**Figure 2B**). In *S.* I 4,[5],12:i:- isolate CQ24NA0063SAL, a 3,088-bp transposition unit (IS*Ecp1*-*bla*_CMY-2_) was inserted into the hypothetical protein gene hypothetical protein gene (*hp1*) with 5-bp DRs (5’-TGGTT-3’). This *bla*_CMY-2_-bearing contig was located on a 96,117-bp IncI1 plasmid pCQ22NA0063SAL-CMY2 (PX317239.1) (**Supplementary Figure 2**), showing 100% coverage and 100% identity with IncI1 plasmid pHUM3355_C (CP093320.1), obtained from an *E. coli* strain HUM3355 isolate from a patient in Botswana.

In three *S.* Thompson isolates, a 4,489-bp transposition unit (IS*Ecp1*-*bla*_CMY-2_-*blc*-*sugE*) was inserted downstream of the plasmid conjugal transfer gene *traA*, generating 5-bp DRs (5’-TGGTT-3’). The *S.* Thompson isolate CQ22WZ0330SAL was found to harbor a 152,834-bp IncC plasmid pCQ22WZ0330SAL-CMY-2 (PX260926.1) that carried this transposition unit (**Supplementary Figure 3A**). This plasmid is closely related (showing 100% coverage and > 99% identity) to the IncC plasmid p846 (CP029249) from a *S.* Thompson clinical isolate in Hefei, China (**Supplementary Figure 3B**).

In *S.* Bovismorbificans a 6,345-bp transposition unit (IS*Ecp1*-*bla*_CMY-2_- *blc*-*sugE*-ΔIS*26*-Δ*hp2*) was inserted downstream of the gene *mprA* (encoding transcriptional repressor protein) with 5-bp DRs (5’-TATTA-3’). We did not locate any transposition unit sharing structural similarity with this one.

A 3,515-bp segment (IS*Kpn26*-ΔIS*Ecp1*-*bla*_CTX-M-64_-*orf477*) was observed in *S.* Typhimurium isolates CQ22WZ0201SAL and CQ22WZ0145SAL (**Figure 2C**). This *bla*_CTX-M-64_-bearing contig shared 100% identity with the corresponding region of plasmid pSG20, which was identified in a *Salmonella* strain SG2 from chicken in China.

In *S.* Kentucky isolate CQ24YZ1359SAL, a 3,577-bp transposition unit (IS*Ecp1*-*bla*_CTX-M-14b_) was inserted upstream of the gene *hp3* with 5-bp DRs (5’-TAGAT-3’) (**Figure 2D**). This *bla*_CTX-M-14b_-bearing contig was 100% identical to the corresponding chromosomal region of *S.* Kentucky strain N16-1393 (CP091999.1), a clinical isolate from Switzerland.

Three different genetic environments of *bla*_CTX-M-65_ were identified in 17 *Salmonella* isolates (**Figure 2E**). In *S.* Infantis isolates CQ23SPB0006SAL and CQ23SPB0007SAL, a 3,873-bp segment (*bla*_CTX-M-65_-IS*903B*-Δ*hp4*-Δ*hp5*) was flanked by inversely oriented IS*26* elements. Downstream of this segment was a 3,310-bp segment IS*Vsa3*-*hp6*-*floR*. This *bla*_CTX-M-65_-bearing contig was 100% identical to the corresponding region of IncFIB plasmid pZ1323HSL0027-2 (CP148798.1). This plasmid was identified in an *S.* Infantis strain Z1323HSL0028 from a human in South Korea.

Fourteen isolates shared an identical *bla*_CTX-M-65_ region (ΔIS*Ecp1*-*bla*_CTX-M-65_-IS*903B*) (**Figure 2E**). A 1,255-bp region, containing two hypothetical protein genes and an incomplete IS*26* (126-bp), was found upstream of the *bla*_CTX-M-65_ region. A 2,243-bp region, containing three hypothetical proteins and an incomplete IS*26* (126-bp), was found upstream of the *bla*_CTX-M-65_ region. This *bla*_CTX-M-65_-bearing contig (5,937-bp) was 100% identical to the corresponding region of IncHI2 plasmid pS304_1 (CP061127.1). This plasmid was identified in a *S.* Typhimurium strain (S304) from a hospital in Hangzhou, China.

In *S.* London isolate CQ23SPB0009SAL, we identified a large novel IS*Ecp1*-mediated transposition unit carrying the *bla*_CTX-M-65_ gene. A 75,302-bp region carrying an IncI plasmid backbone structure was inserted as a composite transposon, which disrupted the hypothetical protein gene *hp7* and generated 5-bp direct repeats (5’-TAATA-3’). This plasmid backbone was closely related to that of the IncI plasmid p3-2 (sharing >99% identity), a plasmid isolated from a patient-derived *Salmonella* strain in Hangzhou, China (**Supplementary Figure 4**).

To better visualize the 75-kb IS*Ecp1*-mediated transposition unit identified in this study. We compared eight complete IS*Ecp1*-mediated transposition units of varying sizes (flanked by 5-bp target site duplications) and their geographic distributions (**Supplementary Figure 5**; **Supplementary Data 4**). Using the seven complete IS*Ecp1*-mediated transposition units from this study as reference sequences, we performed NCBI BLAST searches for highly similar structures (coverage ≥100%, identity ≥99.8%). The most frequently identified group (~2.8 to ~3.1 kb), which corresponds to the typical ISE*cp1*-*bla*_CTX-M-55_/*bla*_CTX-M-65_ structures, was found in 22 countries across Asia, Europe, North America, and Oceania, with China (38/93), Thailand (20/93), and the USA (16/93) being the most frequent. Medium-sized elements (~4.2 to ~4.5 kb) were predominantly identified in China (93/157) and the USA (37/157). Larger elements (~6.3 kb and ~36 kb) were rarely found. The 75-kb transposition unit identified in this study is substantially larger than all previously reported IS*Ecp1*-mediated elements carrying *bla*_CTX-M_/*bla*_CMY-2_, representing the largest such structure documented to date.

### 3.4 Transferability of *bla*_CTX-M_/*bla*_CMY-2_

Conjugation experiments revealed successful transfer of *bla*_CTX-M_/*bla*_CMY-2_ into the recipient in 14 of 36 isolates (38.9%), including the IncC plasmid pCQ22WZ0330SAL-CMY-2 and the IncI1 plasmid pCQ22NA0063SAL-CMY2, conferring CTX resistance (**Supplementary Data 1**).

### 3.5 Phylogenetic analysis of same serotype strains

To determine the genomic relatedness among strains of the same serotype, SNP-based phylogenies were constructed using CSI Phylogeny 1.4. The SNP data are provided in **Supplementary Data 3**. We created maximum-likelihood phylogenetic trees based on SNPs in the core genome for each serotype strain. Closely related *S.* 1,4,[5],12:i:- isolates CQ24YC0426SAL and CQ24YC0423SAL carrying *bla*_CTX-M-55_ were identified from the same hospital, these isolates differed by only 1 SNP (**Figure 3A**). Of the 6 *S.* Goldcoast isolates, three isolates collected from the same hospital in 2024 were clustered into a clade with 0–1 SNP. Isolate CQ24WZ0463SAL from hospital-1 was closely related to isolate CQ24WZ0083SAL from hospital-2, differing by only 3 SNPs (**Figure 3B**).

**Figure 3:**
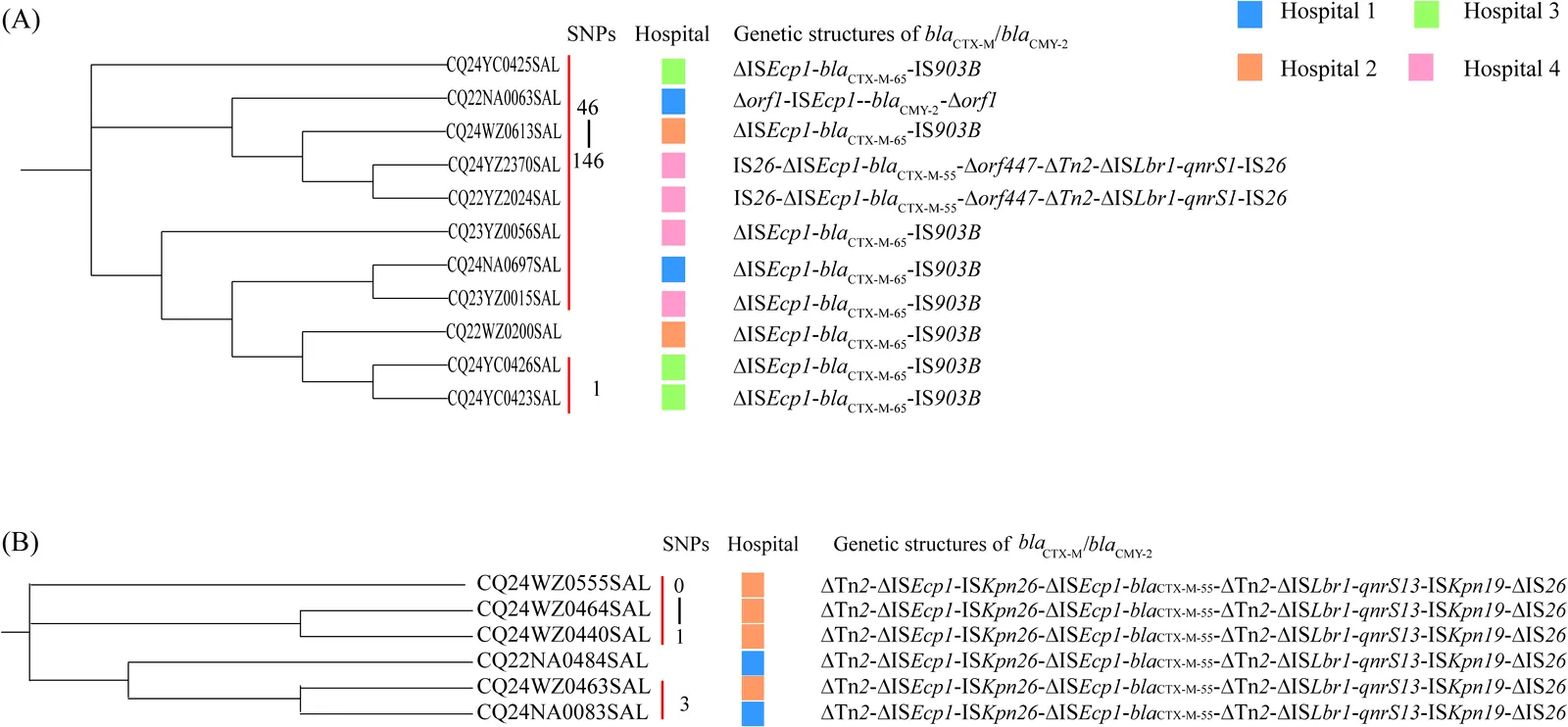
Maximum-likelihood phylogenetic trees based on core-genome SNPs of 11 *S.* 1,4,[5],12:i:- strains **(A)** and 6 *S.* Goldcoast strains **(B)**.

## 3 Discussion

Among 306 Salmonella isolates recovered from patients with diarrhea at seven hospitals in Chongqing during 2022–2024, 36 (11.8%) were confirmed as CTX-resistant and were further characterized in this study. All 36 exhibited resistance to CTX and AMP, and the majority (≥86%) were also resistant to CHL and TET, indicating a high proportion of multidrug-resistant (MDR) phenotypes. Nationwide data from the Chinese Pathogen Identification Network (2017–2023) further showed that the overall resistance level of *Salmonella* in China has reached 73.63%, with domestically observed CIP and CTX resistance exceeding international levels (China CDC Weekly, 2025). The high AMR burden observed in our study is consistent with findings from large-scale genomic surveys. Wang et al. analyzed nearly 8,000 *Salmonella* genomes from China and found that Chinese isolates carried the highest AMR burden globally, with *bla*_CTX-M-65_ and *bla*_CTX-M-55_ being the most frequently detected ESBL variants nationwide (Wang et al., 2023). The temporal dynamics of antimicrobial-resistant *Salmonella* in China further revealed that the prevalence of resistance to critically important antimicrobials, including third-generation cephalosporins and fluoroquinolones, has increased substantially over the past two decades (Wang et al., 2022). Globally, a high-resolution genetic atlas of 208,233 *Salmonella* genomes from 148 countries/regions confirmed that AMR levels have increased across six continents, particularly in serovars such as I 4,[5],12:i:-, and identified antibiotic consumption and socioeconomic factors as key drivers of AMR development (Wang et al., 2025b). Our findings align with this national trend and highlight that CTX-resistant isolates in this regional collection carry an even heavier MDR burden. Here, we provide a detailed characterization of the genetic contexts of *bla*_CTX-M_/*bla*_CMY-2_ in CTX-resistant *Salmonella* from Chongqing, with particular emphasis on the role of IS*Ecp1* in their dissemination. Specifically, the carriage rate of IS*Ecp1* flanking *bla*_CTX-M_/*bla*_CMY-2_ was 94.4%, with diverse transposition units identified adjacent to these resistance genes. As a regional pilot study, our data offer a localized perspective that reinforces the importance of monitoring mobile genetic elements driving cephalosporin resistance at the regional level.

Non-typhoidal *Salmonella* (NTS) from patients with diarrhea represents the most common clinical presentation of NTS infection. The bacteria enter the body through contaminated food or water, colonize the intestinal tract, and invade epithelial cells via virulence factors, triggering localized inflammation and diarrhea. This condition is usually self-limiting. Recent genomic surveillance has uncovered the emergence and spatiotemporal patterns of WHO priority antibiotic resistance in invasive NTS at both the global and national levels (Pei et al., 2026; Wang et al., 2026). These findings underscore the critical threat of antimicrobial resistance in invasive infections, which carry high mortality and require prompt, effective therapy. In contrast, diarrheal NTS (dNTS) imposes a heavier disease burden worldwide and serves as a major reservoir of antimicrobial resistance genes (Wang et al., 2026; WHO, 2022). Therefore, genomic surveillance of dNTS that carry resistance genes and associated mobile genetic elements is equally critical for monitoring the spread of resistance determinants and preventing their potential transmission to more vulnerable populations or invasive lineages.

Although all isolates were resistant to CTX, only 63.89% were resistant to CAZ. This discrepancy can be explained by the substrate preference of CTX-M enzymes. These enzymes typically hydrolyze CTX efficiently but have limited activity against CAZ. An exception exists for certain variants that have acquired enhanced CAZ resistance (Judge et al., 2023). Importantly, all isolates remained fully susceptible to carbapenems (ETP and MEM), CZA, TGC, CT, and AMI. Although carbapenem-resistant *Salmonella* has sporadically emerged in China (Chen et al., 2025), such resistance remains rare, and our data confirm that these last-resort agents are still reliable for treating severe infections caused by CTX-resistant *Salmonella* in Chongqing. However, the high rate of CAZ resistance (63.89%) is clinically concerning because third-generation cephalosporins are often used empirically for suspected *Salmonella* infections before susceptibility results are available.

The high MDR rates were observed in this study—particularly the near-universal resistance to ampicillin and the elevated resistance to CHL (88.9%), TET (86.1%), and CAZ (63.9%). Clinically, these findings suggest that empirical use of these agents should be avoided without susceptibility testing, while carbapenems and tigecycline remain viable last-resort options for severe infections. For surveillance, the high MDR rates and shared co-resistance patterns highlight the importance of incorporating MDR phenotype monitoring into regional surveillance efforts, with a focus on detecting temporal changes that may signal emerging resistance trends.

*S.* I 4,[5],12:i:- was the most frequently identified serotype, accounting for 30.6% (11/36) of the isolates. This monophasic variant of *S.* Typhimurium, which lacks the ability to express the second-phase flagellar antigen, has become a major cause of nontyphoidal *Salmonella* infections worldwide over the past two decades. Consistent with our findings, recent national surveillance data from China (2014–2021) identified *S.* I 4,[5],12:i:- as one of the top five serotypes recovered from sporadic diarrhea cases (Li et al., 2023). Notably, a recent study in Wanzhou District, Chongqing (2022–2024) identified *S.* 1,4,[5],12:i:- as the most frequently identified serotype (23/66, 34.8%), followed by *S.* Typhimurium and *S.* Thompson (Kong et al., 2025). Our results are highly consistent with these local data, further confirming that *S.* I 4,[5],12:i:- has become a dominant serotype among CTX-resistant *Salmonella* in this regional collection.

In addition, *S.* Thompson (3/36, 8.33%) was identified in this study. It is noteworthy that all three *S.* Thompson isolates in our collection carried the AmpC β-lactamase gene *bla*_CMY-2_. These findings align with recent reports from China. Li et al. characterized a multidrug-resistant *S.* Thompson isolate from a retail ready-to-eat pork product, which carried *bla*_CMY-2_, *qnrS1*, and *qepA8* on a conjugative IncA/C plasmid (Li et al., 2022). More recently, Zhang et al. documented the co-location of *bla*_CMY-2_ and plasmid-mediated quinolone resistance (PMQR) genes on IncC plasmids in *S.* Thompson isolates derived from seafood and human patients with diarrhea in Shanghai (Zhang et al., 2024). Similarly, a nationwide genomic tracing study in China (1997–2020) revealed that the rise of *S.* Thompson co-resistant to CIP, CTX, and AZM was driven by IncC plasmids carrying *bla*_CMY-2_, *qnrS1*, *qepA4*, and *mph(A)* (Zhai et al., 2025). These findings suggest that *S.* Thompson may serve as an important reservoir for the dissemination of *bla*_CMY-2_ via mobile genetic elements in China. Our results support this observation and highlight the need for continuous monitoring of *S.* Thompson in clinical settings in Chongqing.

In this study, the ESBL gene *bla*_CTX-M_ was detected in 31 out of 36 isolates (86.1%). Four variants were identified, with *bla*_CTX-M-65_ (17/31, 54.8%) and *bla*_CTX-M-55_ (11/31, 35.5%) being the most frequently detected. This distribution pattern is consistent with recent reports from other regions of China. A study in Hangzhou (2020–2021) reported that among ESBL-positive *Salmonella* isolates, *bla*_CTX-M-65_ and *bla*_CTX-M-55_ were the predominant variants (Yu et al., 2024). Similarly, an investigation in Shenzhen (2020–2022) identified the same three *bla*_CTX-M_ variants (*bla*_CTX-M-14_, *bla*_CTX-M-55_, and *bla*_CTX-M-65_) among CTX-resistant *Salmonella* isolates (Wang et al., 2025a). These findings suggest that *bla*_CTX-M-65_ and *bla*_CTX-M-55_ are frequently detected ESBL genes in clinical *Salmonella* isolates across multiple regions of China.

The AmpC β-lactamase gene *bla*_CMY-2_ was identified in five isolates (13.9%). Notably, all three *S.* Thompson isolates in this study carried *bla*_CMY-2_. A similar phenomenon has been noted in previous studies from China. A nationwide genomic study traced the evolution of *S.* Thompson in China from 1997 to 2020 (Zhai et al., 2025). The study found that *bla*_CMY-2_, together with *qnrS1* and *qepA4*, was located on IncC plasmids. These IncC plasmids were the predominant mobile vectors. They drove the dissemination of multidrug-resistant *S.* Thompson across multiple provinces in China. Additionally, Zhang et al. reported that *bla*_CMY-2_ and PMQR genes were co-located on IncC plasmids. These plasmids were found in *S.* Thompson isolates recovered from seafood and human patients with diarrhea in Shanghai (Zhang et al., 2024). Conjugation experiments further confirmed the co-transfer of these resistance genes. These observations suggest that *S.* Thompson may play a role in the carriage and potential dissemination of *bla*_CMY-2_ in China.

Among the 36 *Salmonella* isolates in this study, a high proportion (26/36, 72.2%) also carried th*e qnrS* gene. Similar co-carriage has been documented in other regions. Liu et al. investigated *Salmonella* isolates from food animals in Shandong Province and reported that among 45 *bla*_CTX-M_-positive isolates, 16 (35.6%) also carried *qnrS* (Liu et al., 2025). In a study of *Salmonella* isolates from broiler and layer chicken farms in Egypt, 170 isolates carried *bla*_CTX-M_, of which 60 (35.3%) also harbored *qnrS* (Elsayed et al., 2024). These co-carriage rates are lower than the 72.2% observed in our clinical isolates, but they demonstrate that the simultaneous presence of ESBL and PMQR genes occurs across different sources, including clinical, food animal, and poultry farm settings.

Notably, we identified one *S.* Montevideo isolate harboring *bla*_CTX-M_/*qnrS*. *S.* Montevideo is a zoonotic serotype associated with international foodborne outbreaks in Europe and North America, but has been rarely reported in China (Gieraltowski et al., 2013; Dominguez et al., 2009; CDC, 2025). A recent genomic study from Shanghai detected two *S.* Montevideo isolates from outpatients in August 2013. These isolates belonged to ST10844 and predated the serotype’s first assignment in EnteroBase (October 2023) by approximately ten years, indicating local circulation since at least 2013 (Lv et al., 2025). Co-carriage of *bla*_CTX-M_/*qnrS* in *S.* Montevideo has been reported in China (Liu et al., 2026). Our study adds to the limited data on this serotype in the Chongqing region. Given the limited data on this serotype, further surveillance is warranted to evaluate its role as a potential reservoir of ESBL and PMQR genes in this regional collection.

In the present study, two distinct plasmid types, IncI1 and IncC, were identified as carriers of the IS*Ecp1*-*bla*_CMY-2_ transposition unit in *Salmonella* isolates from patients with diarrhea in Chongqing. IncI1 plasmids are common among *E. coli* and *Salmonella* from poultry and other avian sources, and have also been increasingly recognized as important vectors of antimicrobial resistance in clinical isolates from human patients, including those with diarrheal infections^[10]^. Phylogenomic analysis has demonstrated that widely occurring *bla*_CMY-2_-carrying IncI1-Iγ plasmids originate from a common ancestor, suggesting a shared evolutionary origin (Castellanos et al., 2019). The high sequence identity (100%) between pCQ22NA0063SAL-CMY2 (PX317239.1) from a patient with diarrhea in Chongqing and pHUM3355_C from an *E. coli* strain isolated from a patient with diarrhea in Botswana suggests the global stability of this IncI1 plasmid backbone, underscoring its potential as an important vector for the dissemination of *bla*_CMY-2_.

In the *S.* Thompson isolate CQ22WZ0330SAL, the *bla*_CMY-2_ gene was carried on a 152,834-bp IncC plasmid pCQ22WZ0330SAL-CMY-2 (PX260926.1). This plasmid showed 100% coverage and >99% identity to the IncC plasmid p846 (CP029249) from a clinical *S.* Thompson isolate in Hefei, China, suggesting that this plasmid backbone may have been horizontally transferred across regions. Nationwide genomic tracing of *S.* Thompson in China (1997–2020) revealed that IncC plasmids are the predominant mobile vectors among CIP, CTX, and AZM-resistant isolates, harboring *qnrS1*, *qepA4*, *bla*_CMY-2_, and *mph(A)* (Zhai et al., 2025). Importantly, the same study documented that closely related and clustered IncC-harboring isolates (0–23 SNPs) have spread across different provinces of China, indicating clonal expansion of these clinically important *S.* Thompson lineages. The high sequence identity between our IncC plasmid and the Hefei-derived IncC plasmid p846, together with the documented clonal spread of IncC-harboring isolates, suggests that both horizontal plasmid transfer and clonal dissemination contribute to the circulation of *bla*_CMY-2_ in clinical *S.* Thompson populations in China.

IS*Ecp1* or incomplete IS*Ecp1* was detected flanking *bla*_CTX-M_/*bla*_CMY_ in 94.4% (34/36) of the isolates. IS*Ecp1* is known to provide promoter sequences for the expression of downstream *bla*_CTX-M_ genes and to facilitate their capture and transposition. A study on ceftriaxone-resistant *S.* Enteritidis in China reported that the dissemination of *bla*_CTX-M_ genes was associated with IS*Ecp1* and IS*26*, and plasmid fusion events involving resistance and virulence plasmids were observed (Yang et al., 2024). Similarly, the emergence of a hybrid IncI1-Iα plasmid-encoded *bla*_CTX-M-101_ in *S.* Enteritidis was attributed to IS*Ecp1*-mediated transposition as the key mechanism for horizontal transfer among plasmids (Qin and Zhang, 2023). In *Klebsiella pneumoniae*, chromosomal integration of *bla*_CTX-M-15_ was largely derived from an IS*Ecp1*-targeting AT-rich locus, and such integration was linked to stable propagation and dissemination in clinical settings (Yoon et al., 2020). Therefore, the high IS*Ecp1* carriage rate in our study further supports an association between IS*Ecp1* and the dissemination of *bla*_CTX-M_/*bla*_CMY-2_ among CTX-resistant *Salmonella* in this regional collection highlighting the need for ongoing surveillance of IS*Ecp1*-mediated transposition events.

Notably, we discovered a novel 75-kb transposition unit, which is substantially larger than previously reported IS*Ecp1*-mediated insertions. Typical IS*Ecp1*-mediated transposition units are much smaller, such as the 2.971-kb IS*Ecp1*-*bla*_CTX-M-15_-Δ*orf477* transposition unit commonly reported. For comparison, an IS*Ecp1*-mediated transposition of a 26.5-kb DNA fragment carrying *bla*_KPC_ has been documented in *Acinetobacter baumannii* (Martínez et al., 2014). More recently, a 36-kb DNA fragment carrying *bla*_CTX-M-32_ was shown to be transferred via IS*Ecp1*-mediated transposition during natural transformation between *A. baumannii* and *Salmonella* (Domingues et al., 2025). Another study reported an IS*Ecp1*-mediated chromosomal integration of a complete IncM1 plasmid-like sequence (carrying *bla*_CTX-M-14_) in *K. pneumoniae* (Hamed et al., 2024). Consequently, such mobilization facilitates the genomic integration and stable propagation of multidrug resistance determinants across different bacterial species. However, conjugation experiments performed in this study did not detect transfer of the 75-kb unit in our study. Nevertheless, this element carries IS*Ecp1* flanks and 5-bp direct repeats—structural signatures of transposition. These features suggest it may act as a large mobile element.

The high carriage rate of IS*Ecp1*-flanked *bla*_CTX-M_/*bla*_CMY-2_ (94.4%) and the structural diversity of the transposition units identified in this study have important implications for regional surveillance strategies. These findings argue for strengthened genomic surveillance in the region. WGS enables more effective tracking of the evolution and dissemination of resistance, as well as resistant clones and mobile elements. The near-universal association of IS*Ecp1* with *bla*_CTX-M_/*bla*_CMY-2_ suggests that resistance dissemination in this region is driven primarily by mobile genetic elements rather than by clonal expansion alone. Surveillance programs should therefore be better positioned to monitor IS*Ecp1*-mediated transposition units as independent indicators, in addition to conventional resistance genes. The structural diversity observed (from ~2.8 kb to 75 kb) underscores the need for WGS-based surveillance to detect emerging variants. Notably, a 75-kb transposition unit was identified in this region. Its structural features suggest a potential to mobilize large DNA segments. These segments may carry multiple resistance determinants. Ongoing genomic surveillance should continue to monitor the transmission and evolution of this unusually large transposition unit.

In addition to IS*Ecp1*, IS*26* was also identified in 28 out of 36 isolates (77.8%) in our study. IS*26* is a well-characterized insertion sequence known to mediate homologous recombination between plasmids and chromosomes, and has been implicated in the formation of compound transposons and the dissemination of multidrug resistance regions in Enterobacteriaceae (Harmer and Hall, 2024). IS26 was frequently found flanking *bla*_CTX-M_/*bla*_CMY-2_ or IS*Ecp1*-mediated transposition units, suggesting that it may cooperate with IS*Ecp1* in shaping the architecture of resistance regions.

In this study, core-genome SNP (cgSNP) typing was used to further elucidate the genetic relatedness among *Salmonella* isolates of the same serotype. Using a threshold of <5 cgSNPs, we identified at least three clusters of closely related isolates, suggesting potential epidemiological links. Most of the closely related isolates were recovered from the same hospital, suggesting potential intra-hospital spread. Notably, several *S.* Goldcoast isolates from two different hospitals differed by only 3 cgSNPs, indicating a possible inter-hospital transmission link. However, direct epidemiological evidence (e.g., patient transfer or environmental contamination) was not available in this study.

However, our study has several limitations. The small sample size (36 isolates from seven hospitals) and lack of denominator data limit assessment of true cefotaxime resistance prevalence. In addition, the 36 isolates were distributed across 13 serotypes, with many represented by only 1–2 isolates, which limits comparisons of resistance patterns across serotypes. Moreover, the use of short-read sequencing in this study resulted in fragmented assemblies for many *bla*_CTX-M_/*bla*_CMY-2_-carrying contigs, preventing definitive assignment to chromosomal or plasmid locations for most of these resistance genes. The cgSNP-based transmission inferences are based solely on genomic relatedness, as direct epidemiological evidenceis not available. Nonetheless, these findings offer useful clues for epidemiological tracing and infection control in this region. Therefore, continuous and expanded surveillance of *Salmonellosis* involving multiple hospitals and a larger number of samples is essential to provide a comprehensive understanding of its epidemiology and antimicrobial resistance pattern.

## 4 Conclusion

In summary, this study provides a comprehensive characterization of the genetic environments of *bla*_CTX-M_/*bla*_CMY-2_ in 36 CTX-resistant *Salmonella* isolates from patients with diarrhea in Chongqing, China (2022–2024). The isolates exhibited high rates of multidrug resistance, with *S.* I 4,[5],12:i:- being the most frequently identified serotype. IS*Ecp1* was identified as the primary insertion sequence associated with the mobilization of *bla*_CTX-M_/*bla*_CMY-2_, present in 94.4% of the isolates. IncHI2A was the most frequently identified plasmid type, while IncI1 and IncC plasmids carrying *bla*_CMY-2_ showed high sequence identity to plasmids from Botswana and other Chinese provinces, suggesting potential inter-regional dissemination. Notably, a novel 75-kb IS*Ecp1*-mediated transposition unit carrying *bla*_CTX-M-65_ and an entire IncI plasmid backbone was found integrated into a hypothetical protein gene of an *S.* London isolate, representing the largest such element reported to date. These findings highlight the frequent association of IS*Ecp1* with cephalosporin resistance genes and suggest a potential for mobilization of large DNA segments. Continuous genomic surveillance of these mobile genetic elements in clinical *Salmonella* isolates is urgently needed to inform infection control strategies and mitigate the further spread of multidrug resistance.

## Data Availability

The data presented in the study are deposited in the NCBI repository (BioProject: PRJNA1332418), and the BioSample accession numbers are listed in **Supplementary Data 1**.

## Supplementary material

Supplementary Figure 1 Comparison of the *bla*_CTX-M-55_ genetic environment in *S.* Montevideo isolate CQ24YC0331SAL with IncHI2 plasmid pAA738 (CP140756.1).

Supplementary Figure 2 Structure of the *bla*_CMY-2_-carrying IncI1 plasmid pCQ22NA0063SAL-CMY2.

Supplementary Figure 3 **(A)** Genetic organization of the *bla*_CMY-2_-carrying IncC plasmid pCQ22WZ0330SAL-CMY-2 (PX260926.1). **(B)** Alignment of pCQ22WZ0330SAL-CMY-2 with the IncC plasmid p846 (CP029249) from a clinical *S.* Thompson isolate in Hefei, China.

Supplementary Figure 4 Structural comparison between the large IS*Ecp1*-mediated transposition unit (75,302-bp) carrying *bla*_CTX-M-65_ in *S.* London CQ23SPB0009SAL and the IncI reference plasmid p3-2.

Supplementary Figure 5 Diversity and geographic distribution of eight types of IS*Ecp1*-mediated transposition units carrying *bla*_CTX-M_/*bla*_CMY-2_. **(A)** Schematic representation of eight IS*Ecp1*-mediated transposition units carrying *bla*_CTX-M_/*bla*_CMY-2_, showing their structural sizes and genetic compositions; **(B–F)** Geographic distribution of the five size variants (~2.8 kb, ~2.9 kb, ~3.1 kb, ~4.2 kb, and ~4.5 kb) of IS*Ecp1*-mediated transposition units. Each map corresponds to one size variant as indicated.
