# Supplementary material for: ISEcp1-associated dissemination of blaCTX-M/blaCMY-2 among cefotaxime-resistant non-typhoidal Salmonella from patients with diarrhea in Chongqing, China, 2022–2024
Source: Front Cell Infect Microbiol. 2026 Sep 28;16:1891482. doi: 10.3389/fcimb.2026.1891482 (PMC13654711; doi:10.3389/fcimb.2026.1891482)
Supplement: Supplementary file 1 [file Table1.docx]

Table S1 The percentage of other ARGs

| ARGs | No. Of ARGs (prevalence, %) | ARGs | No. Of ARGs (prevalence, %) |
| --- | --- | --- | --- |
| *floR* | 30, 83.33% | *arr-3* | 3, 8.33% |
| *tet(A)* | 23, 63.89% | *aadA16* | 2, 5.56% |
| *qnrS1* | 19, 52.78% | *aadA2* | 2, 5.56% |
| *sul2* | 15, 41.67% | *aadA16* | 2, 5.56% |
| *dfrA14* | 15, 41.67% | *aadA22* | 2, 5.56% |
| *strA* | 12, 33.33% | *dfrA27* | 2, 5.56% |
| *strB* | 12, 33.33% | *qnrS2* | 2, 5.56% |
| *aac(3)-IV* | 11, 30.56% | *OqxA* | 2, 5.56% |
| *sul3* | 11, 30.56% | *OqxB* | 2, 5.56% |
| *bla*_TEM-1B_ | 10, 27.78% | *fosA7* | 2, 5.56% |
| *bla*_OXA-10_ | 8, 22.22% | *bla*_DHA-1_ | 1, 2.78% |
| *tet(B)* | 7, 19.44% | *bla*_TEM-32_ | 1, 2.78% |
| *sul1* | 6, 16.67% | *aadA17* | 1, 2.78% |
| *qnrS13* | 5, 13.89% | *aadA7* | 1, 2.78% |
| *arr-2* | 5, 13.89% | *aac(3)-Iid* | 1, 2.78% |
| *cmlA1* | 4, 11.11% | *qnrB* | 1, 2.78% |
| *aadA1* | 3, 8.33% |  |  |

Table S2 Characteristics of *bla*_CTX-M_/*bla*_CMY-2_-carrying contigs in 36 cefotaxime-resistant Salmonella isolates

| Strain | ESBL  genotype | Other resistance genes | Length of *bla*_CTX/CMY_-  contig (bp) |
| --- | --- | --- | --- |
| CQ22NA0063SAL | *bla*_CMY-2_ | _ | 3088 |
| CQ22NA0484SAL | *bla*_CTX-M-55_ | *bla*_TEM-1_, *qnrS13* | 15583 |
| CQ22WZ0200SAL | blaCTX-M-65 | _ | 2376 |
| CQ22WZ0201SAL | blaCTX-M-64 | _ | 3136 |
| CQ22WZ0330SAL | blaCMY-2 | _ | 4489 |
| CQ22YZ2024SAL | blaCTX-M-55 | *qnrS1* | 8993 |
| CQ23RC00148SAL | blaCTX-M-65 | *—* | 2375 |
| CQ23SPB0006SAL | blaCTX-M-65 | *floR* | 9021 |
| CQ23SPB0007SAL | blaCTX-M-65 | *floR* | 9021 |
| CQ23SPB0009SAL | blaCTX-M-65 | *—* | 76767 |
| CQ23WZ0132SAL | blaCMY-2 | — | 4489 |
| CQ23WZ0145SAL | blaCTX-M-64 | — | 3136 |
| CQ23YZ0015SAL | blaCTX-M-65 | — | 2375 |
| CQ23YZ0056SAL | blaCTX-M-65 | — | 2375 |
| CQ24NA0083SAL | blaCTX-M-55 | *bla*_TEM-1_, *qnrS13* | 15583 |
| CQ24NA0653SAL | blaCMY-2 | _ | 8575 |
| CQ24NA0697SAL | blaCTX-M-65 | _ | 2375 |
| CQ24NA0910SAL | blaCTX-M-55 | *qnrS1* | 12838 |
| CQ24SPB0003SAL | blaCTX-M-65 | _ | 2375 |
| CQ24SPB0004SAL | blaCTX-M-65 | _ | 2375 |
| CQ24WZ0440SAL | blaCTX-M-55 | *bla*_TEM-1_, *qnrS13* | 15583 |
| CQ24WZ0463SAL | blaCTX-M-55 | *bla*_TEM-1_, *qnrS13* | 15583 |
| CQ24WZ0464SAL | blaCTX-M-55 | *bla*_TEM-1_, *qnrS13* | 15583 |
| CQ24WZ0555SAL | blaCTX-M-55 | *bla*_TEM-1_, *qnrS13* | 15583 |
| CQ24WZ0575SAL | blaCMY-2 | _ | 4489 |
| CQ24WZ0576SAL | blaCTX-M-65 | _ | 2375 |
| CQ24WZ0598SAL | blaCTX-M-65 | _ | 2375 |
| CQ24WZ0613SAL | blaCTX-M-65 | _ | 2375 |
| CQ24WZ0645SAL | blaCTX-M-55 | *bla*_TEM-1_, *qnrS1* | 12200 |
| CQ24YC0331SAL | blaCTX-M-55 | *aac(3)-IId*, qnrS1 | 13864 |
| CQ24YC0423SAL | blaCTX-M-65 | _ | 2375 |
| CQ24YC0425SAL | blaCTX-M-65 | _ | 2375 |
| CQ24YC0426SAL | blaCTX-M-65 | _ | 2375 |
| CQ24YC0447SAL | blaCTX-M-65 | _ | 2375 |
| CQ24YZ1359SAL | blaCTX-M-14b | _ | 2859 |
| CQ24YZ2370SAL | blaCTX-M-55 | *qnrS1* | 6717 |
